# Circadian *CLOCK* gene polymorphisms in relation to sleep patterns and obesity in African Americans: findings from the Jackson heart study

**DOI:** 10.1186/s12863-017-0522-6

**Published:** 2017-06-23

**Authors:** Pia Riestra, Samson Y Gebreab, Ruihua Xu, Rumana J Khan, Amadou Gaye, Adolfo Correa, Nancy Min, Mario Sims, Sharon K Davis

**Affiliations:** 1 National Human Genome Research Institute Genomics of Metabolic, Cardiovascular and Inflammatory Disease Branch Social Epidemiology Research Unit, National Institutes of Health, 10 Center Drive, Bethesda, MD 20892 USA; 2 Jackson Heart Study, Jackson Medical Mall, 350 West Woodrow Wilson Av., Suite 701, Jackson, MS 39217 USA; 3 Jackson Heart Study, University of Mississippi Medical Center, 350 W Woodrow Wilson Ave, Ste 701, Jackson, MS 39213 USA

**Keywords:** Single nucleotide polymorphism, Obesity, African Americans, CLOCK gene sleep, Body mass index, Adipocytokines

## Abstract

**Background:**

Circadian rhythms regulate key biological processes and the dysregulation of the intrinsic clock mechanism affects sleep patterns and obesity onset. The *CLOCK* (circadian locomotor output cycles protein kaput) gene encodes a core transcription factor of the molecular circadian clock influencing diverse metabolic pathways, including glucose and lipid homeostasis. The primary objective of this study was to evaluate the associations between *CLOCK* single nucleotide polymorphisms (SNPs) and body mass index (BMI). We also evaluated the association of SNPs with BMI related factors such as sleep duration and quality, adiponectin and leptin, in 2962 participants (1116 men and 1810 women) from the Jackson Heart Study. Genotype data for the selected 23 *CLOCK* gene SNPS was obtained by imputation with IMPUTE2 software and reference phase data from the 1000 genome project. Genetic analyses were conducted with PLINK

**Results:**

We found a significant association between the *CLOCK* SNP rs2070062 and sleep duration, participants carriers of the T allele showed significantly shorter sleep duration compared to non-carriers after the adjustment for individual proportions of European ancestry (PEA), socio economic status (SES), body mass index (BMI), alcohol consumption and smoking status that reach the significance threshold after multiple testing correction. In addition, we found nominal associations of the *CLOCK* SNP rs6853192 with longer sleep duration and the rs6820823, rs3792603 and rs11726609 with BMI. However, these associations did not reach the significance threshold after correction for multiple testing.

**Conclusions:**

In this work, *CLOCK* gene variants were associated with sleep duration and BMI suggesting that the effects of these polymorphisms on circadian rhythmicity may affect sleep duration and body weight regulation in Africans Americans.

**Electronic supplementary material:**

The online version of this article (doi:10.1186/s12863-017-0522-6) contains supplementary material, which is available to authorized users.

## Background

Obesity is a complex and multifactorial chronic disorder. It is well known that body weight is strongly influenced by genetic, behavioral and environmental factors [1]. Among the environmental factors related to obesity, great importance has been attributed to changes in eating patterns and physical activity. However, other changes in behavior generated by current lifestyle practices may be associated with obesity, including sleep patterns. In fact, regulation of appetite and food intake is influenced by sleep duration, and sleep restriction, which in turn may contribute to an increase risk of obesity [2]. Common genetic effects were observed between insomnia and both sleepiness and obesity, suggesting shared genetic contributions to these phenomena [3].

In most living organisms, biological processes are orchestrated by 24-h cycles of light and dark that indicate the optimal timing of physiological and behavioral functions. These rhythmic daily physiological adaptations known as ¨circadian rhythms¨ (circa = around; dies = one day) repeated every 24 h are essential to coordinate vital processes including hormone secretion, body temperature regulation and feeding behavior with the exogenous environment [4]. However, circadian rhythms are not merely a response to the environmental 12-h photoperiod but also arise from a endogenous timekeeping mechanism which is composed of circadian clocks, these circadian timers not only include the central or “master clock” located in the suprachiasmatic nucleus (SCN) of the ventral hypothalamus but also peripheral oscillators located in different tissues [5]. This system is emerging as a key and modifiable factor involved in the development of metabolic alterations since disruptions in this system (chronodisruptions), for example such as altered sleep patterns associated with shift working, are associated with metabolic alterations and increased adiposity. Moreover, studies in children and young adults have shown a direct association between fewer hours of sleep and weight gain, and an increased chance of developing obesity in adult life [6, 7]. The core components of the circadian clock mechanism in mammals are genes that encode highly conserved transcription factors and enzymes. The link between circadian rhythmicity and the optimal functioning of relevant metabolic processes has been established at multiple levels, with the discovery that the circadian timekeeping function of the clock genes through transcriptional translational feedback loops is not limited to the SNC but also to most peripheral cells and tissues of the body, including those involved in metabolic homeostasis and feeding behavior such as mediobasal hypothalamus, liver, muscle, and adipose tissue [8, 9]. Recent investigations also revealed the importance of the adipocyte-intrinsic timekeeping mechanism in regulating adipose tissue physiology and consequently glucose and lipid metabolism and adipocytoquine circadian release [10–13].

The transcription factor encoded by the *CLOCK* gene plays a key role in the mammalian circadian system. This transcription factor also contributes to a normal energy metabolism influencing different metabolic pathways such as glucose and lipid metabolism in target organs including muscle, liver and adipose tissue [14]. Thus, some metabolic disorders are frequently associated with mutations in clock related genes explained by an altered expression of these transcriptional regulators which in turn affect key metabolic factors that regulate feeding behavior at the hypothalamic level and energy metabolism in peripheral tissues [15, 16]. Studies have shown that *CLOCK* mutant mice present altered feeding patterns, increased body weight and develop metabolic syndrome accompanied by alterations in lipid and glucose metabolism. These findings support that a tight connection exists between the endogenous circadian system and metabolism [16, 17]. In addition, the CLOCK gene region (4q12) was linked to obesity in a microsatellite-based whole-genome linkage analysis [18]. Single nucleotide polymorphisms (SNPs) in this gene have been related with sleep reduction, adipocytoquine concentrations [19], body mass index (BMI) and energy intake [20]. Garaulet et al. also described an interaction between *CLOCK* SNPs and fatty acids influencing metabolic syndrome traits [21]. Analyses of the human clock genes diversity patterns in terms of environmental adaptation have led to contradictory conclusions, with different studies supporting either the simple effects of gene flow and genetic drift [22] or the influence of natural positive selection based on environmental variables such as seasonal variations in day length or photoperiod across latitudes acting as selective pressure [23, 24]. African Americans an admixed population in terms of genetic ancestry, have a higher prevalence of obesity and metabolic alterations as well as suboptimal sleep durations than other ethnic groups, but to date no studies have evaluated the relationship between *CLOCK* polymorphisms and sleep duration and the presence of obesity in a large sample of African Americans. Thus, the Jackson Heart Study (JHS) may enable us to validate and replicate these findings as well as to investigate whether these health disparities could be in part explained by the differences in genetic associations of the *CLOCK* genes with variables related to body weight regulation.

## Research design and methods

### Study sample

The JHS is a large community-based, prospective cohort study initiated in 2000 to investigate risk factors of heart disease in adult African Americans. The JHS includes a total of 5301 self-reported African Americans, aged 21 to 94 years, residing in three adjacent counties in the Jackson, MS metropolitan area that were recruited, interviewed and examined by certified technicians according to standardized protocols at a baseline exam visit (2000–2004) [25, 26]. The clinic visit included physical examination, anthropometry, survey of medical history, cardiovascular risk factors and collection of blood and urine for biological variables. A total of 3029 (57.1%) participants gave written consent for participation in the genetic analyses and were genotyped using the Affymetrix Genome-Wide Human SNP Array 6.0 (Affymetrix, Santa Clara, CA, USA). The final sample for the present study included 2926 participants (1116 men and 1810 women) who had available *CLOCK* SNPs genotyping information, serum adiponectin and leptin measurements, as well as self-reported sleep quality and quantity data. The study protocol was approved by the institutional review boards of the National Institutes of Health and the participating JHS institutions including the Jackson State University and the University of Mississippi Medical Center, Jackson State University and Tougaloo College.

### Study outcome variables

Weight was measured to the nearest 0.5 kg using a balance scale, in light clothing, without shoes. BMI was calculated as the weight in kilograms divided by the square of height in meters. Fasting (8 h) venous blood samples were drawn from each participant at baseline visit. Serum vials were stored at the JHS central repository in Minneapolis, MN, at −80 °C until assayed. Serum Adiponectin concentrations were measured using an ELISA system (R&D Systems; Minneapolis, MN, USA) [25, 27]. The inter-assay coefficient of variation was 8.8%. Leptin serum concentrations were measured using the Human Leptin RIA kit (LINCO Research, St Charles, MI, USA) with an acceptable coefficient of variation of 10% (22). No biological degradation has been described using stored specimens, indicating a high validity for our measurements.

Sleep duration was defined in hours, based on self-reported responses to the following question: “During the past month, excluding naps, how many hours of actual sleep did you get at night―or during the day, if you work at night―on average?” Perceived quality of sleep (“During the past month, how would you rate your sleep quality overall?”) was a self-rated assessment based on the selection of the following options; “Excellent,” “Very Good,” “Good,” “Fair,” or “Poor.”

### Covariates

The information of the main covariates that are known to influence body weight and serum leptin and adiponectin concentration was collected at baseline. Age was derived from date of birth. Socioeconomic status (SES) was based on self-reported annual household income and divided into three categories (≤$20,000, $20,000 – $50,000, ≥ $50,000). Smoking status was self-reported and defined as current smoker vs. former/never smoker. Alcohol drinking status was also self-reported and defined as “yes” if participant reported ever consuming alcohol and “no” for those reporting never consuming alcohol.

Global European Ancestry estimates for our sample were calculated as previously described [28] using HAPMIX in an analyses supported by the CARe consortium [29–31]. Briefly, For the selection of Ancestry Informative Markers (AIMs) two parental populations that included 1178 European Americans and 756 Nigerians from the Yoruba region were used.. First, using EIGENSOFT [32], a total of 3192 unlinked AIMs were obtained with an allele frequency between parental samples of at least 30%. Next, HAPMIX was run in a mode that assigns European or African ancestry to each allele, thus resolving the local ancestry of each allele when both genotype and local ancestry were heterozygous. Finally, the proportion of the global European ancestry (PEA) for each sample was computed as the average of local ancestry estimates across the genome (scaled to 0.0, 0.5 or 1.0). The proportion of global European ancestry estimates for this sample study had a median of 15.0%.

### SNP selection, genotyping and imputation

With the aim to investigate all the genetic variability in the *CLOCK* gene we used a tag SNP approach for the selection of the genetic variants in the study. This tagging approach was applied on the entire set of common genetic variants in the *CLOCK* gene that included 5 kb upstream of the first exon and 5 kb downstream of the last exon, with minor allele frequency (MAF) ≥1% in Yoruba population (YBI) according to the International HapMap Project (release #24; http://www.hapmap.org). The selection of tagging SNPs was made using the Tagger algorithm available through Haploview [33], considering a pairwise SNP selection with a minimum r^2^ threshold of 0.8, this process resulted in the selection of 23 tagging SNPs for the *CLOCK* gene, with a mean r^2^ of the selected SNPs of 0.969. This selection therefore captures a high degree (over 95%) of the known common variations in this gene. Additional relevant candidate SNPs were selected after reviewing the literature.

Genotype imputation was performed using IMPUTE2 software based on reference phased data from the 1000 Genome Project. Details regarding the generation of the data can be found in the Phase 1 article (The 1000 Genomes Project Consortium, 2012) [34]. Related individuals were removed before the genotype imputation.

Related individuals were removed prior imputation. In the first step of imputation, individuals with pedigree relatedness or cryptic relatedness (pi_hat >0.05) were removed. A subset of random individuals was selected in the second step for estimating recombination and error rates for the total sample, these rates were used to impute all the individuals across the entire reference panel. Results were filtered for imputation at an RSQ less than 0.7 and a MAF threshold of 0.01.

### SNPs quality control

SNP-level quality control metrics were applied prior to downstream analyses. These were: call rate ≥ 95%, minor allele frequency (MAF) ≥1%, Hardy-Weinberg equilibrium (HWE) *p* > 10^−3^, and quality measures for imputed SNPs (r^2^ ≥ 0.3).Additional files 1 Table S1 shows the characteristics, minor allele frequencies and HWE *p*-values for the studied *CLOCK* SNP. All the SNPs presented a high imputation quality (r^2^ > 0.7). Of the 23 SNPs, five were deviated from HWE (*p* < 0.001) and were removed for the analysis. We exclude two variants with a MAF <0.05 (rs17777927 and rs17085885). After the exclusion, a total of 16 SNPs were analyzed in this JHS cohort. The minor allele frequencies for the analyzed SNPs ranged from 5.75% to 37.9%

### Statistical analyses

Statistical Package SAS version 9.3 (SAS Institute Inc., Cary, North Carolina.) was used for descriptive statistical analysis. Participant characteristics were summarized by means and standard deviations for continuous variables and counts and percentages for categorical variables.

The power calculation for testing the associations between CLOCK SNPs and the study outcomes was computed using QUANTO 1.2.3. (https://www.usc.edu/), considering minor allele frequencies and the mean values of BMI and mean adiponectin levels from the JHS study and the effect sizes originally reported by previous studies [35]. The sample size required to detect a 2% of variation in adiponectin levels with an 80% of power and considering a *p*-value of 0.001 is 845. A χ2 goodness-of-fit test was used for testing deviations from Hardy-Weinberg equilibrium for each *CLOCK* SNP considering a significance threshold of *P* > 0.001.

Association analyses between the SNPs and the study phenotypes were performed with PLINK software [36]. Associations between *CLOCK* SNPs and continuous (BMI, adiponectin and leptin serum levels and sleep duration) and categorical outcome variables (sleep quality) categorized as poor and fair vs. good, very good, excellent were tested with separate linear and logistic regression models respectively under an additive genetic model adjusted for age, gender, BMI, SES, smoking status, alcohol consumption and except in the analysis with BMI as an outcome. To control for population stratification, we include the Percentage of European Ancestry (PEA) as a continuous covariate into the models.

Adiponectin and leptin values were log-transformed to obtain better approximations of the normal distribution prior to analysis. A Bonferroni correction was applied to the a priori alpha level of 0.05 and was calculated based on the number of individual SNPs examined (0.05/16). Therefore, a *P*-value threshold of 0.003 was used to determine Bonferroni-corrected statistical significance for *CLOCK* SNPs.

### Multi-marker haplotype-based association testing

Pair-wise linkage disequilibrium (LD) were constructed in the examined region of the *CLOCK* gene using Haploview (Broad Institute) [37]. The haplotype association analyses were also conducted using the haplotype function in PLINK.. Haplotypes with estimated frequency < 5% were excluded from the analysis. In the case of the haplotype analysis that included 5 marker combinations the *P*-value threshold of 0.01 was accepted to determine Bonferroni-corrected statistical significance (0.05/5). Similar to the single variant association analysis, logistic and linear regression models were used to test the associations between the haplotypes and the study outcome variables. All the models were adjusted for age, gender, PEA, BMI, SES, smoking status, alcohol consumption and except in the analysis with BMI as an outcome.

## Results

### Clinical and socio-demographic characteristics of the study participants

Table 1 shows main baseline clinical and socio-demographic characteristics of the study participants according to gender. Of the 2926 self-identified African American participants, 1116 were men and 1810 were women. The median age was 53.67 years for men and 54.88 years for women. BMI was significantly higher in women compared to men (32.28 vs. 30.03, *p* < 0.0001). The prevalence of overweight and obesity was significantly higher in women compared to men (88.95% vs. 82.62%, *p* = 0.001). In comparison with men, women also presented higher serum adiponectin (6.03 μg/mL vs. 4.07 μg/mL, *p* < 0.0001) and leptin concentrations (38.54 ng/mL vs. 11.78 ng/mL, *p* < 0.0001), longer sleep durations (6.48 h vs. 6.31 h, *p* = 0.002) and a higher proportion of annual household income ≤$20,000 (32.03% vs. 19.14%, *p* < 0.0001). Compared with women, men had higher PEA (0.18 vs. 0.17, *p* = 0.006) as well as higher annual household income ≥$50,000 (46.71% vs. 28.50% *p* < 0.0001).

**Table 1:** Baseline clinical and socio-demographic characteristics of the study participants

Variables		Men (*N* = 1116)	Women (*N* = 1810)	
Age (years)		53.67 ± 13.0	54.88 ± 12.71	0.0133
PEA		0.18 ± 0.09	0.17 ± 0.09	0.006
Annual household income				
	<$20,000-	19.14%	32.03%	<.0001
	$20,000–50,000	34.16%	39.48%	
	>$50,000	46.71%	28.50%	
Smoking status				
	Former/never smoker	82.04%	89.04%	<.0001
	Current smoker	18%	10.96%	
Alcohol consumption				
	Former/never drinker	40.63%	59.28%	<.0001
	Current drinker	59.37%	40.72%	
Leptin (ng/mL)		11.78 ± 11.17	38.54 ± 24.87	<.0001
Adiponectin (μg/mL)		4.07 ± 3.39	6.03 ± 4.48	<.0001
BMI (kg/m^2^)		30.03 ± 6.34	32.28 ± 7.80	<.0001
Normal-weight (BMI < 25)		17.38%	11.05%	<.0001
Overweight/Obese (BMI ≥ 25)		82.62%	88.95%	
Sleep duration (hours)		6.31 ± 1.47	6.48 ± 1.49	0.0021
Sleep duration categories				
	Short (≤6 h)	23.98%	26.52%	0.09
	Medium (6-8 h)	70.17%	69.09%	
	Long (>8 h)	5.86%	4.39%	
Sleep quality				
	Poor	11.05%	9.14%	0.10
	Fair	25.41%	22.94%	
	Good	31.82%	34.14%	
	Very good	23.15%	23.48%	
	Excellent	8.56%	10.30%	

Data presented as mean ± SD or as percentages. *P*-values are calculated based on one-way ANOVA for continuous variables and X^2^ test for categorical variables. BMI: body mass index; PEA; proportion of global European ancestry.

### Clinical and socio-demographic characteristics of the study participants according to sleep duration categories

Table 2 shows the participant characteristics by sleep duration categories. Approximately 69.7% (*n* = 2041) of the participants reported sleeping an average of 6–8 h daily, while those with short (<6 h) or long (>8 h) sleep daily comprised 24.9% (*n* = 730) and 5.3% (*n* = 155), respectively. Participants with shorter sleep duration were younger (*p* = 0.0002) and had significantly lower PEA (*p* = 0.0006) compared to those with medium or longer sleep durations. Participants who reported medium or long sleep durations presented significantly lower BMI (31.38, 31.88 vs. 32.61 respectively, *p* = 0.03) and lower consumption of alcohol (48.06, 33.55 vs. 50.27 respectively, *p* = 0.007) compared to those sleeping less than 6 h daily. The percentage of current smokers was lower among participants who reported medium sleep durations compared to those with short or long sleep durations. There were no significant differences in leptin and adiponectin concentration across sleep duration categories.

**Table 2:** Baseline clinical and socio-demographic characteristics of the study participants by sleep duration categories

Variables		Short (< 6 h) 730 (24.95%)	Medium (6–8 h) 2041 (69.75%)	Long (>8 h) 155(5.30%)	*P* value
Age (years)		53.69 ± 12.51	54.37 ± 12.82	58.31 ± 13.6	0.0002
PEA		0.163 ± 0.08	0.178 ± 0.09	0.173 ± 0.08	0.0006
Annual household income					
	<$20,000	26.84%	25.22%	51.52%	<.0001
	$20,000–50,000	38.78%	37.62%	28.03%	
	>$50,000	34.38%	37.16%	20.45%	
Smoking status					
	Former/never smoker	83.33%	87.77%	81.94%	0.003
	Current smoker	16.67%	12.23%	18.06%	
alcohol consumption					
	Former/never drinker	49.73%	51.94%	66.45%	0.007
	Current drinker	50.27%	48.06%	33.55%	
Leptin (ng/mL)		29.61 ± 27.02	27.78 ± 23.51	29.49 ± 24.23	0.185
Adiponectin (μg/mL)		5.16 ± 4.22	5.30 ± 4.16	5.63 ± 4.57	0.42
BMI (kg/m^2^)		32.61 ± 7.75	31.88 ± 7.34	31.38 ± 7.19	0.03
Normal-weight (BMI < 25)		12.88%	13.42%	16.77%	
Overweight/Obese (BMI ≥ 25)		87.12%	86.58%	83.23%	

Data presented as mean ± SD or as percentages.. *P*-values are calculated based on one-way ANOVA for continuous variables and X^2^ for categorical variables. BMI: body mass index; PEA; proportion of global European ancestry

### Associations of CLOCK gene polymorphisms with sleep patterns, BMI, and adipocitoquines related to body weigh regulation

The associations between tagged *CLOCK* gene SNPs and sleep parameters and variables related to body weight regulation are presented in Table 3. The variant rs2070062 was found to be significantly associated with sleep duration, the analysis showed that carriers of the major allele T presented significantly shorter sleep duration compared to non-carriers that reached the significance threshold after Bonferroni correction and adjusting for age, gender, BMI, SES, smoking and PEA (*P* < 0.003). In the case of the variant rs6853192, the T allele showed a nominally significant association with shorter sleep duration as well. However, it failed to reach significance threshold after Bonferroni correction (*p* = 0.02). In addition, we detected nominally significant associations between the *CLOCK* intronic variants rs6820823 and rs3792603 with lower BMI and rs11726609 with higher BMI although none of the variants reached the significance threshold after Bonferroni correction (*p* = 0.01 for rs6820823 and rs3792603: *p* = 0.03 for rs11726609).

**Table 3:** Association of CLOCK SNPs with sleep parameters and variables related to body weight regulation

SNPs		Adiponectin		Leptin		BMI		Sleep Duration		Sleep Quality	
rs	Alleles	β (SE)	*P* value	β (SE)	*P* value	β (SE)	*P* value	β (SE)	*P* value	OR (SE)	*P* value
rs6820823	A/G	−0.018 (0.02)	0.43	−0.017 (0.02)	0.49	−0.018 (0.007)	0.010	0.005 (0.00)	0.50	1.068 (0.07)	0.40
rs17721497	A/T	0.009 (0.03)	0.76	−0.061 (0.03)	0.07	−0.008 (0.009)	0.38	0.007 (0.01)	0.47	1.090 (0.10)	0.41
rs6820119	A/G	−0.027 (0.03)	0.36	0.0067 (0.03)	0.84	0.006 (0.009)	0.52	−0.005 (0.01)	0.60	0.962 (0.10)	0.71
rs3792603	A/G	−0.018 (0.02)	0.43	−0.017 (0.02)	0.49	−0.018(0.007)	0.010	0.005 (0.00)	0.49	1.068 (0.07)	0.40
rs11932595	A/G	−0.014 (0.04)	0.71	0.018 (0.04)	0.68	0.012 (0.012)	0.32	−0.003 (0.01)	0.81	0.899 (0.13)	0.43
rs17085763	T/C	−0.023 (0.03)	0.45	−0.003 (0.03)	0.92	0.006 (0.009)	0.48	−0.005 (0.01)	0.64	0.990 (0.10)	0.93
rs17085780	C/T	0.07 (0.05)	0.18	−0.006 (0.06)	0.91	0.006 (0.016)	0.70	−0.011 (0.01)	0.55	1.193 (0.19)	0.35
rs2070062	T/G	0.007 (0.02)	0.77	−0.009 (0.02)	0.73	0.001 (0.007)	0.83	−0.027 (0.001)	0.003	0.920 (0.08)	0.32
rs7684048	T/C	−0.071 (0.04)	0.08	0.014 (0.04)	0.75	0.0001 (0.01)	0.99	0.007 (0.01)	0.64	1.030 (0.14)	0.83
rs7657206	T/C	−0.110 (0.09)	0.24	0.066 (0.10)	0.53	−0.010 (0.027)	0.69	−0.009 (0.03)	0.77	1.936 (0.37)	0.07
rs11726609	T/A	0.018 (0.01)	0.32	0.026 (0.02)	0.21	0.012 (0.005)	0.03	−0.009 (0.00)	0.1725	0.930 (0.06)	0.27
rs6853192	T/A	−0.0005 (0.03)	0.98	0.044 (0.03)	0.22	0.0096 (0.010)	0.33	−0.027 (0.01)	0.02	1.113 (0.11)	0.34

Alleles listed as major/minor allele; Model adjusted for age, gender, BMI, SES, smoking status, alcohol consumption and PEA: The results of the association are listed for continuous variables as the Beta (standard error) (β (SE)) with the corresponding raw *P*-value. β coefficients represent the change in absolute trait values of each additional risk allele. The results of the association are listed for categorical variables as OR (standard error) (OR (SE)) with the corresponding *P*-value.

SES: socio economic status; BMI: body mass index; PEA; proportion of global European ancestry; The variants rs1801260, rs17085747, rs11931061 and rs12648271 were removed from the table because the software was unable to fit the regression model

### Associations of common five-marker haplotypes with sleep patterns and variables related to body weigh regulation

**Fig. 1:**
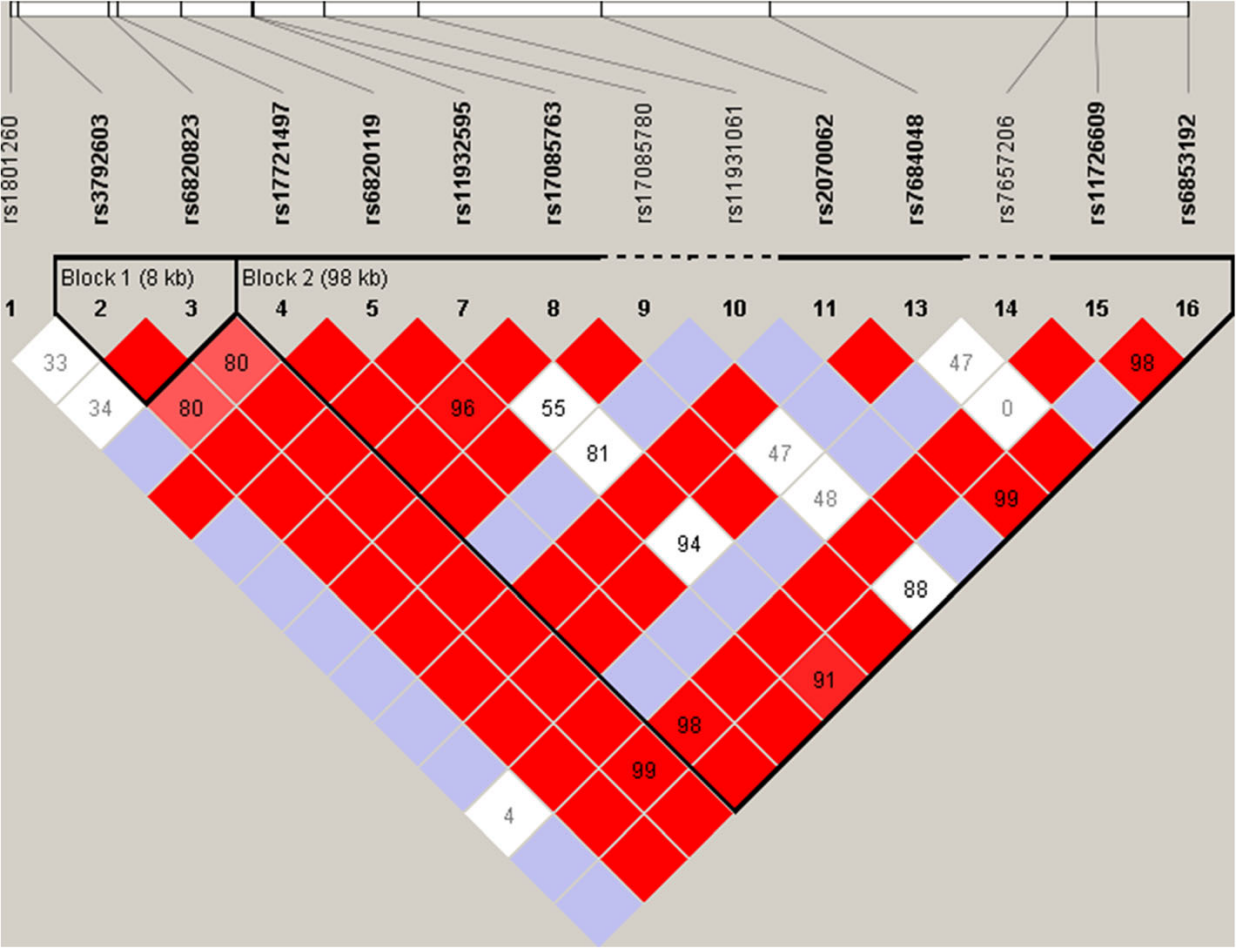
Linkage disequilibrium (LD) plot across the *CLOCK* gene: The horizontal white bar depicts the 117-kb DNA segment of chromosome 4q12 analyzed in the sample. In the LD plot each diamond represents the magnitude of LD for a single pair of markers. Black indicates strong LD (r2 = 1.0); white indicates no LD (r2 = 0); and the gray tones indicate intermediate LD. The numbers inside the diamonds stand for r^2^values

Figure 1 shows the linkage disequilibrium plot for the selected SNPs in the *CLOCK* gene. The plot shows two major haplotype blocks in the *CLOCK* gene in this particular African American cohort.

In order to study the combined effect of *CLOCK* SNPs in the present study, the haplotype analysis only included combinations of those *CLOCK* variants that were significantly or nominally associated with the study outcome variables (i.e. rs2070062; rs6853192; rs3792603; rs6820823 and rs11726609). When considering the five SNPs simultaneously, the five-marker haplotype analysis predicted three common haplotypes (frequency > 5%). The most probable haplotype (22222) had an estimated global frequency of 60%. To assess the association with BMI, sleep duration and quality, adiponectin and leptin serum levels we performed a global test of association across all haplotypes, as well as haplotype-specific association by linear or logistic regression analysis adjusted for age, sex, smoking, SES and PEA.

As shown in Table 4, the five-marker haplotype analysis that included combinations of the five SNPs that were significantly associated with any of the study outcomes did not reveal any significant association between these SNPs and any of the analyzed variables that reached the significance threshold after Bonferroni correction after adjustment for all the covariates.

**Table 4:** Associations between the most common five-marker haplotype combinations in the CLOCK gene with sleep and body weight regulation related variables

		*Adiponectin*			*Leptin*			*BMI*			*Sleep duration*			*Sleep quality*		
Haplotype	F	β	*T*	P*	β	*T*	P*	β	*T*	P*	β	*T*	P*	OR	*T*	P*
22,221 (GGGCA)	0.05	0.004	0.02	0.88	−0.044	1.5	0.22	−0.013	1.5	0.22	0.025	3.8	0.05	0.95	0.25	0.61
22,112 (GGTTA)	0.19	0.079	3.71	0.05	−0.025	0.30	0.57	−0.008	0.41	0.52	−0.008	0.28	0.59	0.95	0.15	0.69
11,222 (AAGCA)	0.08	−0.019	0.30	0.57	0.071	3.44	0.06	0.015	1.7	0.192	0.015	1.27	0.25	1.11	0.96	0.32
12,222(AGGCA)	0.07	0.017	0.43	0.43	0.017	0.48	0.48	0.015	3.91	0.04	−0.0005	0.003	0.95	0.96	0.31	0.57
22,222 (GGGCA)	0.60	−0.024	0.19	0.19	−0.012	0.37	0.54	−0.008	1.74	0.18	−0.010	2.1	0.14	1.02	0.16	0.68

The results of the association are listed for continuous variables as the Beta (standard error) (β (SE)) with the corresponding *P*-value. β coefficients represent the change in absolute trait values of each additional risk allele. The results of the association are listed for categorical variables as OR (standard error) (OR (SE)) with the corresponding P*: adjusted *p*-value. Model adjusted for age, gender, BMI, SES, smoking status, alcohol consumption and PEA. SES: socio economic status; BMI: body mass index; PEA; proportion of global European ancestry. F: haplotype frequency. T: T value from Wald test

## Discussion

In this study we analyzed the associations of genetic variants and five-marker haplotypes of the *CLOCK* gene with sleep duration, sleep quality and factors related to body weight regulation in a large African American sample from the JHS. One of the main findings was the significant association between the T allele of the rs2070062 SNP with shorter sleep duration that remained significant after the adjustment for covariates and correction for multiple testing. Furthermore, we found that carriers of the T allele of the rs6853192 showed a nominally significant association with shorter sleep duration. In addition, the CLOCK variants rs6820823 and rs3792603 were also nominally associated with lower BMI while the rs11726609 appeared to be nominally associated with a higher BMI. These findings are in accordance with previous studies reporting associations between genetic variants in the *CLOCK* gene with energy intake, body weight regulation and sleep and metabolic alterations [19–21, 38].

There is large epidemiologic evidence that has shown a relationship between sleep curtailment and obesity and metabolic alterations [39]. The link between lower BMI with longer sleep durations has been replicated among studies in different populations [6, 7, 40]. Although the question of whether obesity in subjects with short sleep is caused by increased energy intake versus decreased energy expenditure has not been elucidated [39]. In our study, subjects with longer sleep patterns presented a significantly lower BMI which is consistent with previous reports describing a lower BMI linked to longer sleep durations. Additionally, in the last few years, several studies suggested that the genetic contributors of the chronotype or the individual characteristics determining morning or evening preference present an important role in regulating the metabolism. In some of these studies, single nucleotide polymorphisms in *CLOCK* genes were associated with increased obesity and metabolic syndrome incidence [21, 41].

*CLOCK* transcription factor is a master regulator of the rhythmic gene expression of a large number of transcription factors involved in the regulation of numerous circadian physiologic and behavioral functions [42] including sleep-wake cycle and hunger regulation through endocrine signals such as leptin and adiponectin. [43]. To date a number of studies have investigated the relationship between *CLOCK* gene SNPs and adiposity parameters and individual components of the metabolic syndrome, some of these variants have been correlated with BMI, metabolic syndrome [19, 41], energy intake [20] and T2D [38]. Garaulet et al. also described an interaction between *CLOCK* SNPs and fatty acids influencing metabolic syndrome traits. [21]. Obesity is 1.5 times more prevalent in African Americans compared to other US populations. However, this is the first study to explore the association of *CLOCK* SNPs with some components of body weight regulation in this racial group. We observed in our population that the A allele for both *CLOCK* intronic variants rs6820823 and rs3792603 was related to a decreased BMI while the T allele of the rs11726609 was associated with BMI. In this line studies conducted mainly in Caucasian populations were able to find consistent associations of the functional variant 3111 T > C rs1801260 located in the 3′-UTR in combination with a specific haplotype with obesity, metabolic syndrome and the ability to reduce weight in obese subjects [19, 21, 44, 45]. Overweight or obese carriers of the C allele of this polymorphism present more difficulty losing weight compared to TT homozygotes [19, 46] and presented higher BMI along with alteration in the insulin resistance status [47, 48]. Besides the 3111 T > C SNP, the rs1554483, rs6843722, rs6850524, and rs4864548 CLOCK gene variants alone or combined in haplotypes have been linked to the individual susceptibility to obesity [41].

In addition to BMI, we observed a strong novel association between the rs2070062 *CLOCK* SNP and sleep duration in a manner that carriers of the T allele showed a significant reduction in hours of sleep. Although the rs2070062 is an intronic variant with an unknown function, we hypothesized that it could be affecting the alternative splicing of the mRNA or in linkage disequilibrium with other functional variant. To date, GWAS studies identified common SNPs in the CLOCK gene that explain only a 2% of the phenotypic variance in self-report sleep phenotypes. However, a number of studies conducted in European populations have examined the association between *CLOCK* gene variants and sleep patterns especially sleep duration [49, 50]. In a recent meta-analysis including 194 SNPs in 19 candidate clock genes, Allebrandt et al. showed that two SNPs (rs12649507 and rs11932595) located in the intronic region of the *CLOCK* gene were associated with sleep duration [49]. In addition, an important interaction between the rs12649507 and sleep duration on dietary fat intake was described in other work reinforcing the importance of sleep duration on the genetic predisposition to obesity [51]. These findings are in accordance with a recent study examining diabetes in a big Japanese cohort were the C allele of this polymorphism presented higher odds for sleep disorders defined as short sleep durations [48]. However, the replication of these findings in a large sample of European ancestry participants showed no association between the analyzed *CLOCK* variants and sleep duration [50]. A plausible explanation for the failure to replicate the findings is that the authors only include participant with European ancestry when there are significant known differences in the frequency distribution of *CLOCK* gene alleles among populations [22] and the lack of objective sleep duration measurements across studies (all were self-reported). Further studies regarding the potential interactions between restricted sleep and *CLOCK* polymorphisms influencing the development of obesity are needed. This is of importance in African Americans, a population with a high prevalence of obesity related disorders. In addition, the different self-reported methods used among studies for sleep duration assessment or small sample sizes cannot yield conclusive answers.

In summary, we found a novel strong association between the *CLOCK* variant rs207006 and shorter sleep duration that remained significant after multiple testing correction. In addition, the T allele of the rs6853192 showed a nominally significant association with shorter sleep duration. In relation to body weight regulation, the intronic CLOCK SNPs rs6820823 and rs3792603 appeared to be nominally associated with lower BMI while the rs11726609 was nominally associated with higher BMI. These findings point to a genetic component in the link between chronodisruption and the onset of obesity. However, the small effect of these variants needs further exploration in large populations using robust techniques for the assessment of sleep quality and duration phenotypes such as polysomnography and actigraphy.

## Conclusion

In conclusion, in this study conducted in a large African American cohort, we identified significant associations between *CLOCK* SNPs and sleep duration and BMI that suggest a potential effect of CLOCK gene variants in sleep duration and body weight regulation. These findings contribute to the understanding of endogenous circadian disruptions at the molecular level and their link with metabolic alterations. However, these associations need further investigation in multi-ethnic studies that include gene-environment interactions to truly understand the biological differences in the genomic architecture of the circadian system and clock regulation of metabolic functions.

## Additional file

Additional file 1: Characteristics of the selected single nucleotide polymorphisms (SNPs) in the CLOCK gene (DOCX 17 kb)

## Abbreviations

AIMs: Ancestry Informative Markers
BMI: Body Mass Index
CLOCK: Circadian Locomotor Output Cycles Protein Kaput Gene
MAF: Minor Allele Frequency
PEA: Individual Proportions of European Ancestry
SES: Socioeconomic status
SNP: Single Nucleotide Polymorphism

## References

[1] Dubois L, Ohm Kyvik K, Girard M, Tatone-Tokuda F, Perusse D, Hjelmborg J, Skytthe A, Rasmussen F, Wright MJ, Lichtenstein P (2012). Genetic and environmental contributions to weight, height, and BMI from birth to 19 years of age: an international study of over 12,000 twin pairs. PLoS One.

[2] Vorona RD, Winn MP, Babineau TW, Eng BP, Feldman HR, Ware JC (2005). Overweight and obese patients in a primary care population report less sleep than patients with a normal body mass index. Arch Intern Med.

[3] Watson NF, Goldberg J, Arguelles L, Buchwald D (2006). Genetic and environmental influences on insomnia, daytime sleepiness, and obesity in twins. Sleep.

[4] Albrecht U, Eichele G (2003). The mammalian circadian clock. Curr Opin Genet Dev.

[5] Mohawk JA, Green CB, Takahashi JS (2012). Central and peripheral circadian clocks in mammals. Annu Rev Neurosci.

[6] Cappuccio FP, Taggart FM, Kandala NB, Currie A, Peile E, Stranges S, Miller MA (2008). Meta-analysis of short sleep duration and obesity in children and adults. Sleep.

[7] Van Cauter E, Knutson KL (2008). Sleep and the epidemic of obesity in children and adults. European journal of endocrinology/European Federation of Endocrine Societies.

[8] Balsalobre A (2002). Clock genes in mammalian peripheral tissues. Cell Tissue Res.

[9] Yoo SH, Yamazaki S, Lowrey PL, Shimomura K, Ko CH, Buhr ED, Siepka SM, Hong HK, Oh WJ, Yoo OJ (2004). PERIOD2::LUCIFERASE real-time reporting of circadian dynamics reveals persistent circadian oscillations in mouse peripheral tissues. Proc Natl Acad Sci U S A.

[10] Kalsbeek A, Fliers E, Romijn JA, La Fleur SE, Wortel J, Bakker O, Endert E, Buijs RM (2001). The suprachiasmatic nucleus generates the diurnal changes in plasma leptin levels. Endocrinology.

[11] Gavrila A, Peng CK, Chan JL, Mietus JE, Goldberger AL, Mantzoros CS (2003). Diurnal and ultradian dynamics of serum adiponectin in healthy men: comparison with leptin, circulating soluble leptin receptor, and cortisol patterns. J Clin Endocrinol Metab.

[12] Kalra SP, Bagnasco M, Otukonyong EE, Dube MG, Kalra PS (2003). Rhythmic, reciprocal ghrelin and leptin signaling: new insight in the development of obesity. Regul Pept.

[13] Vieira E, GR E, Figueroa AL, Aranda G, Momblan D, Carmona F, Gomis R, Vidal J, Hanzu FA (2014). Altered clock gene expression in obese visceral adipose tissue is associated with metabolic syndrome. PLoS One.

[14] Yang X, Downes M, Yu RT, Bookout AL, He W, Straume M, Mangelsdorf DJ, Evans RM (2006). Nuclear receptor expression links the circadian clock to metabolism. Cell.

[15] Froy O: The circadian clock and metabolism. Clinical science (London, England: 1979) 2011, 120(2):65–72. 10.1042/CS20100327 20929440

[16] Turek FW, Joshu C, Kohsaka A, Lin E, Ivanova G, McDearmon E, Laposky A, Losee-Olson S, Easton A, Jensen DR *et al*: Obesity and metabolic syndrome in circadian Clock mutant mice. Science (New York, NY) 2005, 308(5724):1043–1045. 10.1126/science.1108750 PMC3764501 15845877

[17] Patel SR, Blackwell T, Redline S, Ancoli-Israel S, Cauley JA, Hillier TA, Lewis CE, Orwoll ES, Stefanick ML, Taylor BC *et al*: The association between sleep duration and obesity in older adults. International journal of obesity (2005) 2008, 32(12):1825–1834. 10.1038/ijo.2008.198 PMC2605208 18936766

[18] Deng HW, Deng H, Liu YJ, Liu YZ, Xu FH, Shen H, Conway T, Li JL, Huang QY, Davies KM (2002). A genomewide linkage scan for quantitative-trait loci for obesity phenotypes. Am J Hum Genet.

[19] Garaulet M, Sanchez-Moreno C, Smith CE, Lee YC, Nicolas F, Ordovas JM (2011). Ghrelin, sleep reduction and evening preference: relationships to CLOCK 3111 T/C SNP and weight loss. PLoS One.

[20] Garaulet M, Lee YC, Shen J, Parnell LD, Arnett DK, Tsai MY, Lai CQ, Ordovas JM (2010). Genetic variants in human CLOCK associate with total energy intake and cytokine sleep factors in overweight subjects (GOLDN population). European journal of human genetics: EJHG.

[21] Garaulet M, Lee YC, Shen J, Parnell LD, Arnett DK, Tsai MY, Lai CQ, Ordovas JM (2009). CLOCK genetic variation and metabolic syndrome risk: modulation by monounsaturated fatty acids. Am J Clin Nutr.

[22] Ciarleglio CM, Ryckman KK, Servick SV, Hida A, Robbins S, Wells N, Hicks J, Larson SA, Wiedermann JP, Carver K (2008). Genetic differences in human circadian clock genes among worldwide populations. J Biol Rhythm.

[23] Cruciani F, Trombetta B, Labuda D, Modiano D, Torroni A, Costa R, Scozzari R (2008). Genetic diversity patterns at the human clock gene period 2 are suggestive of population-specific positive selection. European journal of human genetics: EJHG.

[24] Forni D, Pozzoli U, Cagliani R, Tresoldi C, Menozzi G, Riva S, Guerini FR, Comi GP, Bolognesi E, Bresolin N (2014). Genetic adaptation of the human circadian clock to day-length latitudinal variations and relevance for affective disorders. Genome Biol.

[25] Taylor HA (2005). The Jackson heart study: an overview. Ethnicity & disease.

[26] Carpenter MA, Crow R, Steffes M, Rock W, Heilbraun J, Evans G, Skelton T, Jensen R, Sarpong D (2004). Laboratory, reading center, and coordinating center data management methods in the Jackson heart study. Am J Med Sci.

[27] Bidulescu A, Liu J, Musani SK, Fox ER, Samdarshi TE, Sarpong DF, Vaccarino V, Wilson PW, Arnett DK, Din-Dzietham R (2011). Association of adiponectin with left ventricular mass in blacks: the Jackson heart study. Circulation Heart failure.

[28] Riestra P, Gebreab SY, Xu R, Khan RJ, Bidulescu A, Correa A, Tekola-Ayele F, Davis SK (2015). Gender-specific associations between ADIPOQ gene polymorphisms and adiponectin levels and obesity in the Jackson heart study cohort. BMC medical genetics.

[29] Price AL, Tandon A, Patterson N, Barnes KC, Rafaels N, Ruczinski I, Beaty TH, Mathias R, Reich D, Myers S (2009). Sensitive detection of chromosomal segments of distinct ancestry in admixed populations. PLoS Genet.

[30] Patterson N, Hattangadi N, Lane B, Lohmueller KE, Hafler DA, Oksenberg JR, Hauser SL, Smith MW, O'Brien SJ, Altshuler D (2004). Methods for high-density admixture mapping of disease genes. Am J Hum Genet.

[31] Keating BJ, Tischfield S, Murray SS, Bhangale T, Price TS, Glessner JT, Galver L, Barrett JC, Grant SF, Farlow DN (2008). Concept, design and implementation of a cardiovascular gene-centric 50 k SNP array for large-scale genomic association studies. PLoS One.

[32] Price AL, Patterson NJ, Plenge RM, Weinblatt ME, Shadick NA, Reich D (2006). Principal components analysis corrects for stratification in genome-wide association studies. Nat Genet.

[33] de Bakker PI, Ferreira MA, Jia X, Neale BM, Raychaudhuri S, Voight BF (2008). Practical aspects of imputation-driven meta-analysis of genome-wide association studies. Hum Mol Genet.

[34] Duan Q, Liu EY, Auer PL, Zhang G, Lange EM, Jun G, Bizon C, Jiao S, Buyske S, Franceschini N *et al*: Imputation of coding variants in African Americans: better performance using data from the exome sequencing project. Bioinformatics (Oxford, England) 2013, 29(21):2744–2749. 10.1093/bioinformatics/btt477 PMC3799474 23956302

[35] Hong EP, Park JW (2012). Sample size and statistical power calculation in genetic association studies. Genomics & informatics.

[36] Purcell S, Neale B, Todd-Brown K, Thomas L, Ferreira MA, Bender D, Maller J, Sklar P, de Bakker PI, Daly MJ (2007). PLINK: a tool set for whole-genome association and population-based linkage analyses. Am J Hum Genet.

[37] Barrett JC, Fry B, Maller J, Daly MJ: Haploview: analysis and visualization of LD and haplotype maps. Bioinformatics (Oxford, England) 2005, 21(2):263–265. 10.1093/bioinformatics/bth457 15297300

[38] Corella D, Asensio EM, Coltell O, Sorli JV, Estruch R, Martinez-Gonzalez MA, Salas-Salvado J, Castaner O, Aros F, Lapetra J (2016). CLOCK gene variation is associated with incidence of type-2 diabetes and cardiovascular diseases in type-2 diabetic subjects: dietary modulation in the PREDIMED randomized trial. Cardiovasc Diabetol.

[39] Lucassen EA, Rother KI, Cizza G (2012). Interacting epidemics? Sleep curtailment, insulin resistance, and obesity. Ann N Y Acad Sci.

[40] Watson NF, Harden KP, Buchwald D, Vitiello MV, Pack AI, Weigle DS, Goldberg J (2012). Sleep duration and body mass index in twins: a gene-environment interaction. Sleep.

[41] Sookoian S, Gemma C, Gianotti TF, Burgueno A, Castano G, Pirola CJ (2008). Genetic variants of clock transcription factor are associated with individual susceptibility to obesity. Am J Clin Nutr.

[42] Menet JS, Pescatore S, Rosbash M (2014). CLOCK:BMAL1 is a pioneer-like transcription factor. Genes Dev.

[43] Scheer FA, Morris CJ, Shea SA: The internal circadian clock increases hunger and appetite in the evening independent of food intake and other behaviors. Obesity (Silver Spring, Md) 2013, 21(3):421–423. 10.1002/oby.20351 PMC3655529 23456944

[44] Scott EM, Carter AM, Grant PJ: Association between polymorphisms in the Clock gene, obesity and the metabolic syndrome in man. International journal of obesity (2005) 2008, 32(4):658–662. 10.1038/sj.ijo.0803778 18071340

[45] Garaulet M, Corbalan MD, Madrid JA, Morales E, Baraza JC, Lee YC, Ordovas JM: CLOCK gene is implicated in weight reduction in obese patients participating in a dietary programme based on the Mediterranean diet. International journal of obesity (2005) 2010, 34(3):516–523. 10.1038/ijo.2009.255 PMC4426985 20065968

[46] Garaulet M, Esteban Tardido A, Lee YC, Smith CE, Parnell LD, Ordovas JM: SIRT1 and CLOCK 3111T> C combined genotype is associated with evening preference and weight loss resistance in a behavioral therapy treatment for obesity. International journal of obesity (2005) 2012, 36(11):1436–1441. 10.1038/ijo.2011.270 PMC4428942 22310473

[47] Garcia-Rios A, Gomez-Delgado FJ, Garaulet M, Alcala-Diaz JF, Delgado-Lista FJ, Marin C, Rangel-Zuniga OA, Rodriguez-Cantalejo F, Gomez-Luna P, Ordovas JM (2014). Beneficial effect of CLOCK gene polymorphism rs1801260 in combination with low-fat diet on insulin metabolism in the patients with metabolic syndrome. Chronobiol Int.

[48] Uemura H, Katsuura-Kamano S, Yamaguchi M, Arisawa K, Hamajima N, Hishida A, Kawai S, Oze I, Shinchi K, Takashima N (2016). Variant of the CLOCK circadian regulator (CLOCK) gene and related haplotypes are associated with the prevalence of type 2 diabetes in the Japanese population. Journal of diabetes.

[49] Allebrandt KV, Teder-Laving M, Akyol M, Pichler I, Muller-Myhsok B, Pramstaller P, Merrow M, Meitinger T, Metspalu A, Roenneberg T (2010). CLOCK gene variants associate with sleep duration in two independent populations. Biol Psychiatry.

[50] Lane JM, Tare A, Cade BE, Chen TH, Punjabi NM, Gottlieb DJ, Scheer FA, Redline S, Saxena R (2013). Common variants in CLOCK are not associated with measures of sleep duration in people of european ancestry from the sleep heart health study. Biol Psychiatry.

[51] Dashti HS, Follis JL, Smith CE, Tanaka T, Cade BE, Gottlieb DJ, Hruby A, Jacques PF, Lamon-Fava S, Richardson K (2015). Habitual sleep duration is associated with BMI and macronutrient intake and may be modified by CLOCK genetic variants. Am J Clin Nutr.

