# Supplementary material for: Circadian CLOCK gene polymorphisms in relation to sleep patterns and obesity in African Americans: findings from the Jackson heart study
Source: BMC Genet. 2017 Jun 23;18:58. doi: 10.1186/s12863-017-0522-6 (PMC5481932; doi:10.1186/s12863-017-0522-6)
Supplement: Additional file 1: — Characteristics of the selected single nucleotide polymorphisms (SNPs) in the CLOCK gene (DOCX 17 kb) [file 12863_2017_522_MOESM1_ESM.docx]

S1: Characteristics of the selected single nucleotide polymorphisms (SNPs) in the *CLOCK* gene.

| ***CLOCK* SNPs** | **Other Name in the Literature** | **Location on Chromosome 4 (NCBI 36)^(a)^** | **r^2 (b)^** | **HWE**  **P- value** | **Major Allele** | **Minor Allele** | **MAF** |
| --- | --- | --- | --- | --- | --- | --- | --- |
| rs1801260 | 3111 T/C | 55435202 | 0.99364 | 0.8894 | A | G | 16.79% |
| rs3792603 |  | 55435891 | 0.87129 | 0.9035 | A | G | 5.75% |
| rs6820823 |  | 55444209 | 0.99254 | 0.8276 | A | G | 35.90% |
| rs17721497 |  | 55445081 | 0.9957 | 0.1911 | A | T | 10.32% |
| rs17777927 |  | 55446466 | 0.73704 | 0.5248 | C | G | 1.36% |
| rs17085747 |  | 55452677 | 0.98439 | 0.9779 | T | C | 7.07% |
| rs11932595 |  | 55457430 | 0.9821 | 0.682 | A | G | 36.61% |
| rs17085763 |  | 55457526 | 0.99138 | 0.7304 | T | C | 37.90% |
| rs2140076 |  | 55458376 | 1.00002 | 0.0001 | A | T | 26.40% |
| rs17085780 |  | 55463976 | 0.99863 | 0.2846 | C | T | 14.80% |
| rs11931061 |  | 55472626 | 0.99395 | 0.6682 | A | G | 36.80% |
| rs9997288 |  | 55481060 | 0.99971 | 0.0001 | C | T | 17.11% |
| rs2070062 |  | 55489431 | 0.99873 | 0.5114 | T | G | 16.84% |
| rs12648271 |  | 55501955 | 0.89755 | 0.9705 | G | C | 7.06% |
| rs7684048 |  | 55504877 | 0.96927 | 0.5915 | T | C | 6.08% |
| rs13132420 |  | 55526646 | 0.99662 | 0.0001 | G | A | 19.69% |
| rs7657206 |  | 55531992 | 0.99453 | 0.5472 | T | C | 11.64% |
| rs11726609 |  | 55534702 | 0.99988 | 0.5267 | T | A | 27.52% |
| rs17085885 |  | 55535730 | 0.95927 | 0.1207 | T | C | 3.10% |
| rs4864546 |  | 55537960 | 0.98518 | 0.0001 | G | A | 34.93% |
| rs6853192 |  | 55543087 | 0.9836 | 0.1267 | T | A | 37.60% |
| rs2412651 |  | 55536094 | 0.92703 | 0.0001 | T | C | 1.46% |
| rs6820119 |  | 55450851 | 0.9898 | 0.7413 | T | C | 9.26% |

**^(a)^** Position based on NCBI Build 36: MAF: minor allele frequency; **^(b)^** r^2^ refers to the measurement imputation quality of the SNPs. HWE: [Hardy Weinberg equilibrium](https://www.google.com/search?espv=2&biw=1280&bih=627&q=hardy+weinberg+equilibrium&spell=1&sa=X&ei=0REPVK35DI_lsATR14KwCg&ved=0CBoQvwUoAA)
